# Supplementary material for: Low-Dose-Rate Radiation-Induced Secretion of TGF-β3 Together with an Activator in Small Extracellular Vesicles Modifies Low-Dose Hyper-Radiosensitivity through ALK1 Binding
Source: Int J Mol Sci. 2022 Jul 24;23(15):8147. doi: 10.3390/ijms23158147 (PMC9332371; doi:10.3390/ijms23158147)
Supplement: Supplementary file 1 [file ijms-23-08147-s001.zip › SUPP~QWS.pdf]

# Low dose rate radiation induced secretion of TGF-β3 together with an activator in small extracellular vesicles modifies low dose hyper-radiosensitivity through ALK1 binding

## 1. Supplementary figures

| Marker | 1 | 2 | Marker | 1 | 2 | Marker | 1 | | Marker | 1 | 2 |
|---|---|---|---|---|---|---|---|---|---|---|---|
| *CD9* | ■ | □ | *VCP* | ■ | ■ | *HSPA1A* | □ | □ | *HIST2H4A* | ■ | ■ |
| *HSPA8* | ■ | ■ | *TPI1* | ■ | ■ | *GNAI2* | □ | □ | *GNB1* | □ | □ |
| *PDCD6IP* | ■ | ■ | *PPIA* | ■ | ■ | *ANXA1* | □ | □ | *UBA1* | ■ | ■ |
| *GAPDH* | ■ | ■ | *MSN* | □ | □ | *RHOA* | □ | ■ | *THBS1* | ■ | ■ |
| *ACTB* | ■ | ■ | *CFL1* | ■ | ■ | *MFGE8* | □ | □ | *RAN* | ■ | ■ |
| *ANXA2* | ■ | ■ | *PRDX1* | ■ | ■ | *PRDX2* | ■ | ■ | *RAB5A* | □ | □ |
| *CD63* | □ | □ | *PFN1* | ■ | ■ | *GDI2* | ■ | □ | *PTGFRN* | ■ | ■ |
| *SDCBP* | ■ | □ | *RAP1B* | ■ | ■ | *EHD4* | ■ | □ | *CCT5* | ■ | ■ |
| *ENO1* | □ | □ | *ITGB1* | ■ | ■ | *ACTN4* | ■ | □ | *CCT3* | ■ | ■ |
| *HSP90AA1* | ■ | ■ | *HSPA5* | ■ | ■ | *YWHAB* | ■ | ■ | *BSG* | ■ | □ |
| *TSG101* | ■ | □ | *SLC3A2* | ■ | ■ | *RAB7A* | ■ | ■ | *AHCY* | ■ | ■ |
| *PKM* | □ | □ | *HIST1H4A* | ■ | ■ | *LDHB* | □ | □ | *RAB5B* | ■ | □ |
| *LDHA* | □ | □ | *GNB2* | □ | □ | *GNAS* | □ | □ | *RAB1A* | □ | □ |
| *EEF1A1* | □ | ■ | *ATP1A1* | ■ | ■ | *TFRC* | ■ | ■ | *LAMP2* | ■ | □ |
| *YWHAZ* | ■ | ■ | *YWHAQ* | ■ | ■ | *RAB5C* | ■ | □ | *ITGA6* | ■ | □ |
| *PGK1* | ■ | ■ | *FLOT1* | □ | □ | *ARF1* | ■ | □ | *HIST1H4B* | ■ | ■ |
| *EEF2* | ■ | ■ | *FLNA* | ■ | ■ | *ANXA6* | □ | □ | *GSN* | ■ | ■ |
| *ALDOA* | ■ | ■ | *CLIC1* | ■ | ■ | *ANXA11* | □ | □ | *FN1* | ■ | ■ |
| *HSP90AB1* | ■ | ■ | *CDC42* | ■ | ■ | *ACTG1* | □ | □ | *YWHAH* | ■ | ■ |
| *ANXA5* | □ | □ | *CCT2* | ■ | ■ | *KPNB1* | □ | □ | *TUBA1A* | □ | □ |
| *FASN* | ■ | ■ | *A2M* | □ | ■ | *EZR* | ■ | ■ | *TKT* | ■ | ■ |
| *YWHAE* | ■ | ■ | *YWHAG* | ■ | ■ | *ANXA4* | □ | □ | *TCP1* | ■ | □ |
| *CLTC* | ■ | □ | *TUBA1B* | □ | □ | *ACLY* | □ | □ | *STOM* | □ | □ |
| *CD81* | □ | □ | *RAC1* | ■ | □ | *TUBA1C* | ■ | ■ | *SLC16A1* | □ | □ |
| *ALB* | □ | □ | *LGALS3BP* | □ | ■ | *RAB14* | □ | □ | *RAB8A* | □ | □ |

**Figure S1. List of the 100 proteins most frequently detected in small extracellular vesicles (sEVs)** [1], and their detection in two separate experiments (each with three biological replicates) of sEVs from low dose rate (LDR) irradiated and unirradiated T-47D cells: ■ – detected, □ - not detected.

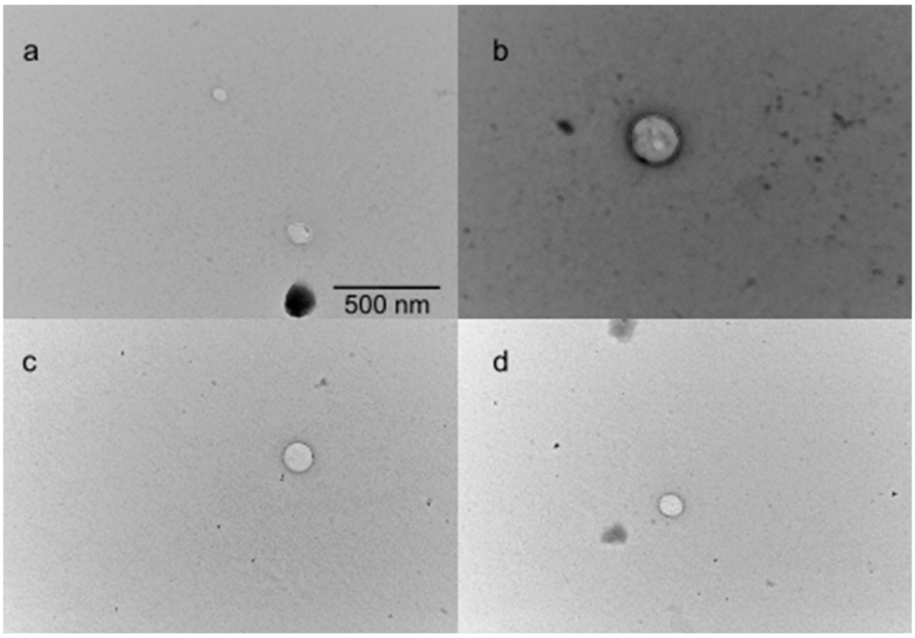

**Figure S2. Transmission electron microscope (TEM) images of extracellular vesicles** isolated from LDR primed (a,b) and unirradiated (c,d) T-47D cells. The shape and size of the isolated particles are consistent with that of sEVs.

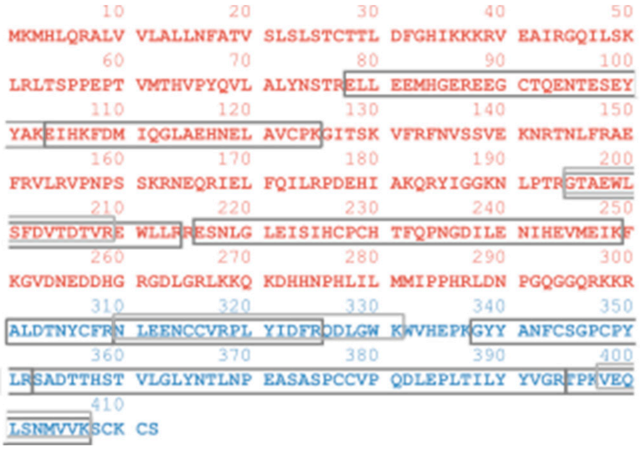


**Figure S3.** Peptide sequence of TGF-β3 (blue) with the latency-associated peptide (LAP) (red). Rectangles mark the peptide sequences that were detected in tandem mass spectrometry (MS/MS) analysis of sEVs from LDR primed and unirradiated cells, in either of two separate experiments , each with three biological replicates.

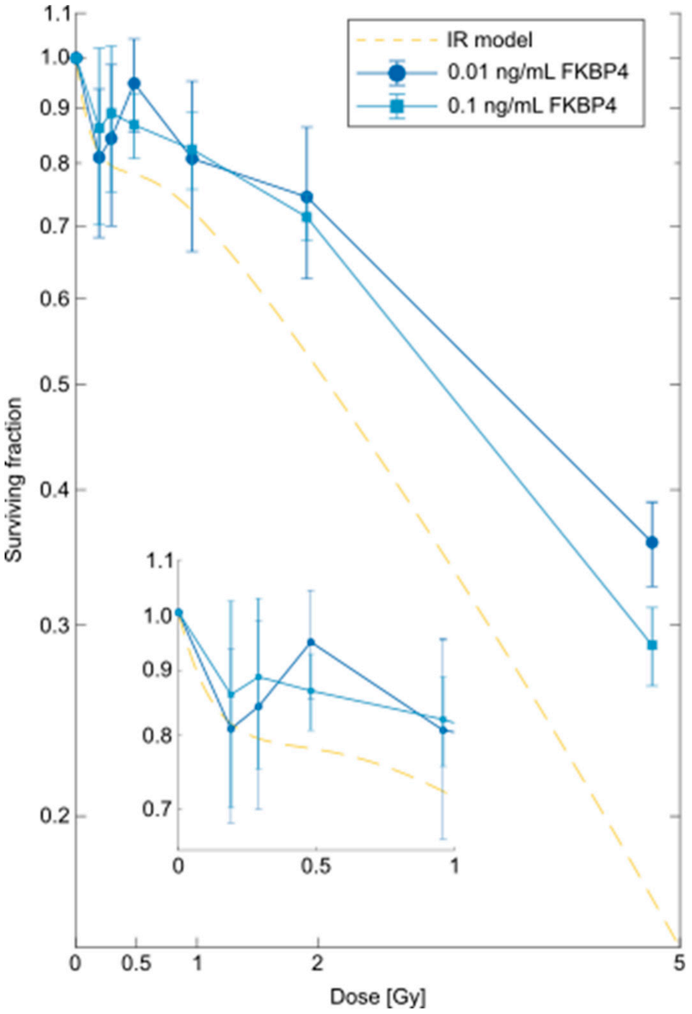

**Figure S4. Survival of T-47D cells after pretreatment with Peptidyl-prolyl cis-trans isomerase FKBP4 (FKBP4).** Addition of 0.01 ng/mL (●) or 0.1 ng/mL (■) recombinant FKBP4 to the medium of T-47D cells 48 hours before challenge $\gamma$-irradiation did not remove hyper-radiosensitivity (HRS) from the cells. IR model= induced repair model[2]-fit for untreated T-47D cells. Surviving fractions are given as error-weighted averages of three separate experiments, each with five biological replicates. Error bars: standard error of the mean. Note that the surviving fractions were calculated relative to the plating efficiency of control cells, which were also exposed to the pretreatments.


[1] Keerthikumar, S.; Chisanga, D.; Ariyaratne, D.; Al Saffar, H.; Anand, S.; Zhao, K.; Samuel, M.; Pathan, M.; Jois, M.; Chilamkurti, N.; et al. ExoCarta: A Web-Based Comendium of Exosomal Cargo. *J Mol Biol*, **2017**, *428* (4), 688–692. https://doi.org/10.1016/j.jmb.2015.09.019.

[2] Marples, B.; Joiner, M. C. The Response of Chinese Hamster V79 Cells to Low Radiation Doses: Evidence of Enhanced Sensitivity of the Whole Cell Population. *Radiat. Res.*, **1993**, *133* (1), 41–51. https://doi.org/10.2307/3578255.
